# Editorial

**DOI:** 10.1080/26415275.2020.1831296

**Published:** 2020-10-14

**Authors:** Anne Peutzfeldt, Jon E. Dahl

Dear Reader, 

Last summer *Biomaterial Investigations in Dentistry* instigated a ‘Best Young Author of the Year’ award. The award is presented to a first author who at the time of submission of his/her manuscript is within 10 years of completing his/her last terminal degree (PhD, DDS, DMD, MD, etc.), and who is of Nordic nationality or has conducted his/her research in a Nordic country (limited geographically by the statutes of the ACTA Odontologica Scandinavica Society). Eligible manuscripts are evaluated based on the following criteria: originality of study, suitability of study design and clarity of manuscript and is accompanied by a prize of 2.000 Euro.

It is with great pleasure that we announce the recipient of the 2019–2020 ‘Best Young Author of the Year’ award: Dr. Ketil Hegerstrøm Haugli of Oslo Metropolitan University who receives the award for his highly original and well-structured manuscript: ‘Ion release from three different dental alloys – effect of dynamic loading and toxicity of released elements’ published online on April 24, 2020. The manuscript was co-authored by Morten Syverud and Jan Tore Samuelsen and selected from a pool of four eligible candidates. We congratulate Dr. Hegerstrøm Haugli and encourage eligible authors to sign up as candidates for the 2020–2021 award.


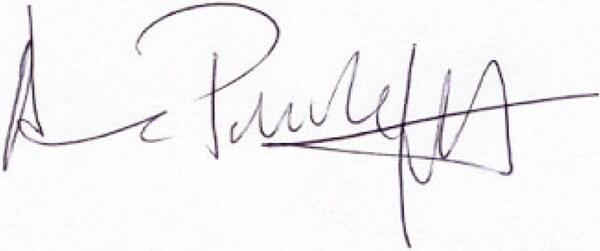


Anne Peutzfeldt

Editor-in-Chief

anne.peutzfeldt@sund.ku.dk


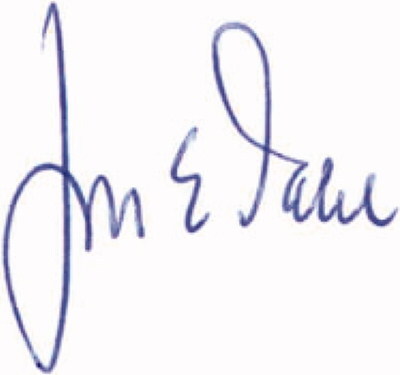


Jon E. Dahl

Associate Editor

